# TYK2 in Cancer Metastases: Genomic and Proteomic Discovery

**DOI:** 10.3390/cancers13164171

**Published:** 2021-08-19

**Authors:** Dana C. Borcherding, Kevin He, Neha V. Amin, Angela C. Hirbe

**Affiliations:** 1 Division of Oncology, Department of Internal Medicine, Washington University School of Medicine, St. Louis, MO 63110, USA; bdana@wustl.edu (D.C.B.); kevinh@wustl.edu (K.H.); amin.n@wustl.edu (N.V.A.); 2 Siteman Cancer Center, Washington University School of Medicine, St. Louis, MO 63110, USA

**Keywords:** tyrosine kinase-2, TYK2, cancer, metastasis, genomics, proteomics, transcriptomics, JAK, STAT

## Abstract

**Simple Summary:**

Cancer deaths are predominantly due to metastases rather than the primary tumors, and thus there is an urgent need for the discovery of more effective drug therapies for metastatic cancer. Recent genomics, transcriptomics, and proteomics studies have identified tyrosine kinase 2 (*TYK2*) as an oncogene that is frequently mutated or overexpressed in many types of cancer and metastases. A member of the Janus kinase (JAK) family, TYK2 mediates the signals of numerous cytokines involved in immune and inflammatory signaling. In cancer cells, activation of TYK2 can lead to decreased cell death as well as increased cell growth and invasion. Multiple drugs that specifically block TYK2 or JAKs are currently FDA-approved or in clinical trials. In this review, we provide an overview of the screening, molecular, and animal studies that have characterized the role of TYK2 in cancer and metastases, and the potential of TYK2 inhibitors as effective cancer therapies.

**Abstract:**

Advances in genomic analysis and proteomic tools have rapidly expanded identification of biomarkers and molecular targets important to cancer development and metastasis. On an individual basis, personalized medicine approaches allow better characterization of tumors and patient prognosis, leading to more targeted treatments by detection of specific gene mutations, overexpression, or activity. Genomic and proteomic screens by our lab and others have revealed tyrosine kinase 2 (*TYK2*) as an oncogene promoting progression and metastases of many types of carcinomas, sarcomas, and hematologic cancers. TYK2 is a Janus kinase (JAK) that acts as an intermediary between cytokine receptors and STAT transcription factors. TYK2 signals to stimulate proliferation and metastasis while inhibiting apoptosis of cancer cells. This review focuses on the growing evidence from genomic and proteomic screens, as well as molecular studies that link TYK2 to cancer prevalence, prognosis, and metastasis. In addition, pharmacological inhibition of TYK2 is currently used clinically for autoimmune diseases, and now provides promising treatment modalities as effective therapeutic agents against multiple types of cancer.

## 1. Genomics, Transcriptomics and Proteomics in Target Discovery: TYK2 in Cancer

Revolutionary advancements in bioinformatics techniques and computational data analysis within the last decade have transformed modern cancer diagnostics and therapeutics [1]. Innovations in next-generation sequencing (NGS) have allowed large-scale genomic and transcriptomic characterization, leading to the detection of novel genetic alterations in numerous types of cancer [1,2]. High throughput proteomics utilizing protein microarrays and mass spectrophotometry have similarly led to the discovery of proteins with aberrant expression or kinase activity in cancer development and progression [3].

Genomic, transcriptomic and proteomic assays have led to identification of tyrosine kinase 2 (*TYK2*) mutations, fusion proteins and expression changes in a variety of hematological cancers, carcinomas and soft-tissue sarcomas [4]. TYK2 is an approximately 134 kDa protein identified in 1990 as the first member of the Janus kinase (JAK) family, which includes non-receptor tyrosine kinases that mediate cytokine signaling [5,6]. In humans, the *TYK2* gene is found on chromosome 19, and is ubiquitously expressed at varying levels throughout the body [7]. Originally known for its immune modulatory role, *TYK2* is now emerging as an oncogene that could serve as both a prognostic biomarker and a promising drug target in cancer [8]. In this review, we discuss the history of TYK2 with an emphasis on the genomic, proteomic, and transcriptomic screens that have led to characterization of the role of TYK2 in cancer and metastases.

### 1.1. TYK2 in Carcinomas and Sarcomas

While activating mutations in other members of the JAK family have long been known to be tumorigenic, it has only been in the last 10 years that a series of screening studies have shown the involvement of *TYK2* as an oncogene driving cancer development and metastases [9]. Early proteomic analyses first reported the role of TYK2 as a biomarker in breast, cervical, colorectal and prostate cancers (Table 1) [10,11,12,13]. A dissociable antibody microarray (DAMA) staining screen of hundreds of proteins, a technique that combines immunostaining and protein microarrays, found that TYK2 protein levels were elevated in breast cancer compared to normal breast cell-lines [10]. Likewise, proteomics using two-dimensional (2D) gel electrophoresis followed by mass spectrometry showed increased TYK2 protein expression in squamous cervical cancer tissue [11]. Similarly, in colorectal cancer cells, high resolution mass spectrophotometry identified TYK2 as a phosphorylation target of hepatocyte growth factor (HGF), which stimulates proliferation in these cells [12]. Proteomic phosphotyrosine peptide enrichment and quantitative mass spectrometry identified several activated kinases, including TYK2 (Y^292^) and its downstream target signal transducer and activator of transcription 3 (STAT3) (Y^705^), in metastatic castration-resistant prostate cancer [13]. In addition, RNA sequencing (RNA-seq) of the transcriptome revealed that *TYK2* and *JAK3* mRNA levels were significantly increased in stomach adenocarcinoma, and both proteins were found to be prognostic biomarkers [14].

Genomic screens have also implicated *TYK2* as a pro-survival gene in soft-tissue sarcomas (Table 1). Our laboratory utilized NGS and identified activating *TYK2* mutations in malignant peripheral nerve sheath tumors (MPNST), an aggressive subtype of sarcomas associated with the Neurofibromatosis type 1 (NF1) cancer predisposition syndrome [36]. Subsequent genetic knockdown of *TYK2* in MPNST cell lines resulted in decreased tumor growth and increased cell death [20]. Additionally, genetic knockdown of *Tyk2* in murine MPNST cells resulted in decreased tumor burden in subcutaneous tumors and metastatic tumor models [20]. In line with these studies, genomic NGS profiling of over 100 patients with multiple types of advanced recurrent, metastatic or refractory sarcomas found that they harbored mutations in *TYK2*, *JAK1*, *JAK2*, and *JAK3* [21].

### 1.2. TYK2 in Hematological Cancers

Hematological cancers, malignancies of the blood that arise from immune cells or the bone marrow, include leukemia, lymphoma, and multiple myeloma. *JAK2*, and to a lesser extent *JAK1* and *JAK3*, play a major oncogenic role in multiple types of leukemia, often undergoing gain-of-function (GOF) mutations and translocations [37,38]. *TYK2* GOF point mutations occur less frequently in leukemia, although many single nucleotide polymorphisms (SNPs) in the coding region of the *TYK2* gene have been described, with over 100 nonsynonymous mutations associated with a number of diseases (Table 1) [39]. Acute lymphoblastic leukemia (ALL) is a subset of leukemia associated with chromosomal rearrangements or genetic mutations that encode for transcription factors that drive cancer progression [40]. At the transcriptome level, an RNA interference (RNAi) screen engaging short hairpin RNA (shRNA) against over 1700 genes in T-cell acute lymphoblastic leukemia (T-ALL) cell lines found that TYK2 promotes cell growth and survival downstream of IL-10, through STAT1 and BCL2 signaling, while TYK2 inhibitors decreased cell proliferation [18]. Sequencing revealed gain-of-function (GOF) *TYK2* point mutations in the FERM, JH2, and kinase domains [18]. In pediatric ALL patients, whole exome sequencing identified *TYK2* mutations in the JH2 pseudokinase domain (G761V and P760L) that promote TYK2 autophosphorylation, activate STAT1/3/5, and are suggested to contribute to the development of leukemia [23,24]. Another subtype, Philadelphia chromosome (Ph)-like ALL, contains multiple kinase-activating alterations in two-thirds of patients, including activation of TYK2, as determined by a NGS genomic profiling of 1725 patients [25,26]. Furthermore, high-throughput sequencing of the kinase domains of tyrosine kinase genes identified a SNP, rs2304255 (TYK2-G363S), in the TYK2 kinase domain that was associated with acute myeloid leukemia (AML), although it is unclear as to the impact of this mutation on TYK2 function [27].

Additionally, four TYK2 fusion proteins have been identified in several hematologic cancers using genomic and transcriptomic sequencing [17,19,25]. In a T-cell lymphoma cell-line, whole-transcriptome screening found a TYK2 fusion protein with nucleophosmin (NPMI), NPMI-TYK2, which includes the kinase domain and a partial pseudokinase domain of TYK2 [19]. The NPMI-TYK2 fusion protein contains a constitutively active TYK2, leading to constitutively phosphorylated STAT1, 3 and 5 [19]. Numerous genomic rearrangements were discovered in human anaplastic large cell lymphoma (ACLC) patients with an RNAseq screen, including two fusions of TYK2 to nuclear factor of kappa light polypeptide gene enhancer in B cells 2 (NFκB2) or poly(A) binding protein cytoplasmic 4 (PABPC4) [17]. Cells with the NFκB2-TYK2 fusion protein exhibited constitutively active TYK2, JAK2, and STAT3, resulting in greater colony forming capacity [17]. Correspondingly, TYK2 is highly expressed in lymphomas including ALCL, and activated TYK2 leads to increased ALCL cell survival, which is mediated through STAT1/3 and the anti-apoptotic protein MCL-1 [41]. In the Ph-like ALL genomic screen, the v-myb avian myeloblastosis viral oncogene homolog (MYB) was merged with TYK2 to form MYB-TYK2 [25]. While this fusion protein includes the TYK2 kinase domain and part of the pseudokinase domain, the study did not assess activation of TYK2 and downstream STATs [25].

### 1.3. Other Actions of TYK2 in Cancer

Similar to what is seen with other genes, the role of *TYK2* in malignancy is complex and likely is cell type and context dependent. In some settings, *TYK2* appears to play a role in suppressing tumor growth, and a few studies have reported lower TYK2 expression or loss-of-function (LOF) mutations associated with cancer development or progression [15,28,42,43]. In a proteomics screen for tyrosine kinase variants, multiple brain and hematopoietic cancer cell lines harbored an inactivating *TYK2* splice variant, E971fsX67 [15]. Mice with knockout of *Tyk2* were more susceptible to xenograft tumor growth and metastasis of breast cancer 4T1 cells, and *Tyk^-/-^* mice also developed leukemia and lymphoma at an increased rate, both likely due to defective tumor immunosurveillance [42,43]. Tumor immunosurveillance is the process of the host immune system identifying and destroying malignant or pre-cancerous cells [44]. Amplicon-based NGS revealed *TYK2* variants with catalytic LOF in 25% of B-cell ALL (B-ALL) patients, as well as lower *TYK2* gene expression overall in B-ALL [28]. These LOF *TYK2* variants failed to phosphorylate STAT3 in cells *in vitro*, and further support the immunosurveillance role of TYK2 in cancer [28]. However, the intracellular mechanisms by which TYK2 deficiency within cancer cells may promote tumorigenesis in a cell autonomous manner under certain circumstances remains unclear.

A computational analysis found that the *TYK2* rs34536443 variant (P1104A) conferred increased cancer risk, and this mutation was subsequently detected in several cancers, including MPNST, breast cancer, colon cancer, stomach cancer and AML [27,36,45]. Located within the activation loop of the highly conserved kinase domain, the P1104A mutation is predicted to cause activation of the catalytic domain [36,45]. Functional studies found that immune cells with the *TYK2* P1104A variant exhibited impaired auto-phosphorylation in response to ATP or IFN-α [39]. Nevertheless, TYK2 P1104A transduced signaling of IFN-α/β, IL-6 and IL-10 to phosphorylate downstream STATs, suggesting that pairing with a catalytically competent JAK is sufficient for cytokine signaling [39]. In addition, the *TYK2* P1104A variant corresponded to TYK2 overexpression in MPNST tumors [20]. Thus, the *TYK2* P1104A variant may drive carcinogenesis through relative changes in the different TYK2-mediated cytokine signaling pathways and downstream STAT-induced gene transcription.

## 2. TYK2 Signaling: Intermediary of Cytokine Signaling and STATs

The JAK family of non-receptor tyrosine kinases is composed of TYK2 and JAK1-3 [46]. TYK2 and JAK1-3 associate with the type I and type II cytokine receptor superfamily, heterodimeric or multimeric receptors without intrinsic kinase activity [47]. TYK2 mediates signal transduction for many cytokines, including interferons (IFN) and interleukins (IL), through association with five receptor chains: IFN-α/β receptor 1 (IFNAR1), IL-12 receptor-β1 (IL-12Rβ1), IL-10 receptor β (IL-10Rβ), IL-13 receptor α (IL-13Rα) and gp130 [48]. When an extracellular ligand binds to and activates its receptor, conformational changes in the transmembrane receptors bring JAKs close together, where they are activated by auto- or trans-phosphorylation and subsequently phosphorylate intracellular tyrosine residues on the receptors [49]. TYK2 heterodimerizes with JAK1 or JAK2, but not JAK3, depending on the cytokine and receptor complex [50]. Activated JAKs then recruit and phosphorylate signal transducers and activators of transcription (STAT), which has seven family members (STAT1, STAT2, STAT3, STAT4, STAT5A, STAT5B, and STAT6) [51]. TYK2 specifically transduces activation of STAT1, STAT3 and STAT5A/B. Phosphorylated STATs then homo- or hetero-dimerize, allowing translocation to the nucleus where STAT dimers bind on the promoters of target genes to induce transcription.

### 2.1. TYK2 Structure and Post-Translational Modifications

**Figure 1:**
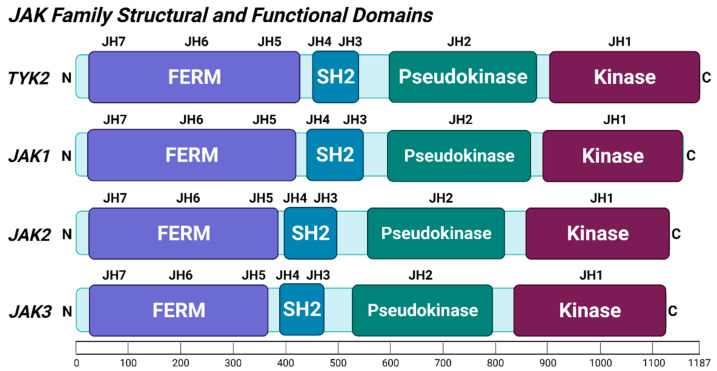
Schematic diagram of the structural and functional domains of the TYK2 and JAK1-3 proteins. TYK2/JAK1-3 consist of four structural domains (FERM, SH2, pseudokinase, and kinase domains) that overlap with seven JAK homology (JH1-7) domains. Scale bar shows amino acid (aa) range (0-1187 aa) from the N-terminus to the C-terminus. Illustration created with BioRender.com (accessed 5 August, 2021).

In vertebrates, JAK1-3 and TYK2 are highly conserved proteins with seven homology domains (JH1-JH7). These form four structural domains: (1) the four-point-one, ezrin, radixin, moesin (FERM) homology domain (part of JH4 and JH5-JH7), (2) src-homology 2 (SH2) domain (JH3 and part of JH4), (3) pseudokinase (kinase-like) domain (JH2), and (4) kinase domain (JH1) (Figure 1) [47,52]. The N-terminal FERM domain facilitates interaction of TYK2/JAK1-3 with the intracellular tails of receptors, while the adjacent SH2 domain is responsible for binding to receptors [53,54]. Various mutations to the catalytically inactive pseudokinase domain can result in either increased or decreased kinase activity [4]. Thus, the JH2 pseudokinase domain functions as a negative regulator of kinase activity in the absence of receptor activation, and conversely, to communicate the signal from the ligand-activated receptor to the JH1 kinase domain [55]. The catalytically active kinase domain on the C-terminal end of TYK2 contains two adjacent tyrosine residues (Y1054 and Y1055) in its activation loop that are auto-/trans-phosphorylated by ligand-binding induced conformational changes in its receptors [47,56]. TYK2 also contains several other phosphorylation sites located throughout all four domains, including tyrosine residues (Y292, Y433, Y827, Y884, and Y1145) and serine residues (S491, S499) [53,57]. Further post-translational modifications of TYK2 besides phosphorylation have not been widely studied.

In a negative feedback loop, STATs induce transcription of suppressor of cytokine signaling (SOCS) genes [58]. SOCS1-7 proteins function as E3 ubiquitin ligases to target JAKs for ubiquitination and degradation via the proteasome [59,60]. In addition, SOCS1 and SOCS3 can bind to the JH1 domain of JAKs to inhibit kinase activity [61]. TYK2 signaling is also inhibited through de-phosphorylation by protein tyrosine phosphatase 1B (PTP1B), src homology 2 domain containing protein tyrosine phosphatase-1 (SHP1), and CD45 [62,63,64]. Additionally, a protein chaperone, heat-shock protein 90 (HSP90), has been shown to interact with and stabilize TYK2 and other JAKs in cancer cells, and thus is a promising therapeutic target [36,65].

### 2.2. Interferons

**Figure 2:**
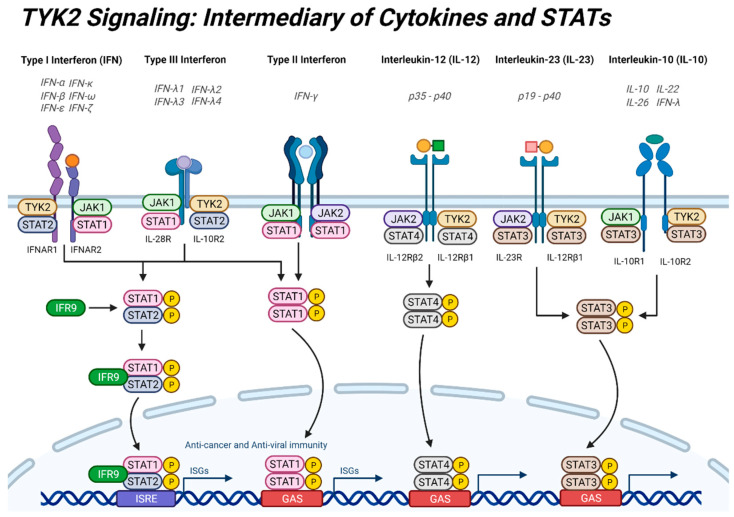
Overview of TYK2 signaling pathways. TYK2 and JAK1/2 interact with cytokine receptors to transduce cytokine signaling through activation of STATs and induction of target gene expression. Cytokine ligands, e.g., interferons (IFN) and interleukins (IL), bind to their respective receptors, leading to auto- or trans-phosphorylation of JAKs/TYK2 and receptor phosphorylation. Signal transducers and activators of transcription (STAT) are recruited and phosphorylated. Activated STATs then homo- or hetero-dimerize and are translocated to the nucleus, where STAT dimers bind to IFN-stimulated response elements (ISRE) or interferon-γ activated sequences (GAS) promoter elements to induce transcription of target genes. Downstream of Type I/III IFNs, STAT1/2 complex with the cofactor interferon regulatory factor 9 (IRF9). Illustration created with BioRender.com.

The earliest studies on TYK2 signaling found it to be an intermediate in the IFNα and IFNβ pathways (Figure 2) [66]. Subsequently, TYK2 was shown to primarily be involved in the signaling of type I and type 2 IFNs [67,68]. The type I IFN family is a large group of mainly IFNα subtypes and IFNβ, as well as IFNε, IFNκ, IFNω, and IFNζ, and these cytokines are involved in anti-viral immunity and anti-cancer immunity [69,70,71,72]. The ubiquitously expressed type I IFNs bind to the IFNAR2 chain that is associated with JAK1, which then recruits the IFNAR1 chain associated with TYK2 [73]. Activated TYK2/JAK1 then typically phosphorylates a STAT1 and STAT2 dimer, and to a lesser extent STAT1 homodimers [74]. Other STATs (STAT3, STAT4, and STAT6) can also transduce type I IFN signaling in some conditions and cell-types [70]. The STAT1/STAT2 dimer complexes with the cofactor interferon regulatory factor 9 (IRF9) to bind to IFN-stimulated response elements (ISRE), while the STAT1/STAT1 homodimer binds to interferon-γ activated sequences (GAS) in promoter regions of IFN-targeted genes [72,73]. Type I IFNs stimulate transcription of over 1000 genes implicated in regulation of inflammatory and immune functions, including in cancer [72].

TYK2 also associates with the IL-10R2 chain which, in combination with the JAK1-associated IL-28R chain, mediates signal transduction of type III IFNs through STAT1/STAT2 heterodimers and IRF9 [75,76]. The type III IFN family includes IFNλ1 (IL-29), IFNλ2 (IL-28A), IFNλ3 (IL-28B) and IFNλ4, which can also induce phosphorylation of the other STATs [77,78]. IFNλs are thought to be responsible for initial host responses against minor infections and epithelial layer damage [78]. Large-scale systemic infections subsequently illicit the stronger type I IFN defense [70]. However, activation of type I IFNs can lead to a systemic overactive immune response or inflammation [79].

### 2.3. Interleukins

The IL-12 family and the IL-10 family of cytokines also utilize TYK2 signal transduction in a balance of immunomodulatory and inflammatory responses. Members of the IL-12 family (IL-12 and IL-23) bind to JAK-associated IL-12Rβ2 or IL-23R chains, respectively, which heterodimerizes with the TYK2-associated IL-12Rβ1 chain to activate primarily STAT4 and STAT3, respectively (Figure 2) [50]. STAT1 and STAT5 can also be activated depending on cell context [80]. IL-12 and IL-23 have counteracting functions in immune function, inflammation and cancer development [81]. IL-12 is important in cell-mediated immunity, while IL-23 promotes inflammation [82].

IL-10 family members are essential anti-inflammatory cytokines with immunosuppressive actions that promote epithelial homeostasis and barrier function in response to infection or inflammatory conditions, while IL-10 deficiency is involved in autoimmune disorders [76]. However, IL-10 has opposing immune stimulatory actions depending on cell type and circumstance [83]. The IL-10 family of cytokines include IL-10, IL-22, IL-26 and IFNλs, which bind to their specific JAK1-associated receptor chains and to the TYK2-associated IL-10R2 chain (IL-10Rβ), leading to phosphorylation of STAT3 homodimers, with a minor component through STAT1 and STAT5 [47,84].

Other cytokines that signal through TYK2 include those that bind to the gp130 receptor subunit, e.g., IL-6, IL-11, IL-27, oncostatin M (OSM), cardiotrophin-1 (CT-1), and leukemia inhibitory factor (LIF). However, TYK2 is redundant with other JAKs in these pathways. A full list of cytokines and receptors reported to signal through TYK2 have been reviewed previously [44,47].

## 3. Immune Modulatory and Inflammatory Effects of TYK2

Immune surveillance against cancer cells and chronic inflammation both influence the microenvironment surrounding tumors throughout their development and metastasis [85]. Inflammatory cytokine signaling can stimulate cancer cell proliferation, migration, and invasion [86]. The family of JAK kinases transduce cytokine-derived signals needed for a functional immune response [7]. Indeed, TYK2 was originally recognized for its roles in immunity and inflammation, with TYK2 primarily mediating the effect of cytokine signaling pathways vital for adaptive and innate immune responses [53]. Given the establishment of *TYK2* as a susceptibility gene in auto-inflammation, immunodeficiency and other immune responses as well as in cancer [39,87,88], the prospect of therapies targeting TYK2 in immunodeficiency and autoimmune diseases exists.

TYK2 deficiency, often via LOF mutations, is frequently involved in severe immunodeficiency. Clinical appearances of TYK2 deficiency are typically mycobacterial and/or viral infections, caused by impaired responses to IL-12 and IFN-α/β [89]. STAT3 and TYK2 signaling alterations are associated with Hyper IgE syndrome (HIES), a rare immunodeficiency characterized by elevated serum immunoglobulin E (IgE), skin inflammation, and recurrent skin and lung infections [90,91]. Similarly, mutations in *STAT3* are associated with the autosomal dominant form of HIES, which also features skeletal, connective tissue and pulmonary abnormalities [92]. The recessive form, characterized by viral infections and neurologic complications, has been linked to TYK2 deficiency; however, the genetic cause is still under investigation [91,93,94]. Several reports describe patients with non-functional TYK2 that have exhibited features of HIES. One patient with complete TYK2 deficiency suffered from frequent bacterial and viral infections as well as HIES-like disease [95]. Two other patients with nonsense *TYK2* mutations displayed characteristics of HIES, but did not experience significant viral or bacterial infections [96,97]. However, seven other TYK2-null patients exhibited viral or mycobacterial infections through diminished IFN-α, IFN-β, and IL-12 signaling, without signs of HIES [89]. In mice, elimination of *Tyk2* produces immunological dysfunctions making the mice highly susceptible to infections and some tumors [68,98,99]. Thus, it is debated whether loss of TYK2 primarily results in viral/mycobacterial infections and constitutes a separate disorder from HIES [89].

While TYK2 deficiency is accompanied by recurrent viral and bacterial infections, elevated TYK2 activity is also connected with complications of viral infections. A large GWAS analysis reported *TYK2* as a genetic locus (rs74956615) associated with severity of COVID-19 disease caused by the novel SARS-CoV-2 virus, with high *TYK2* gene expression found in critically ill COVID-19 patients [29]. Several JAK inhibitors, e.g., ruxolitinib (JAK1/2), baricitinib (JAK1/2), and tofacitinib (JAK1/2/3, TYK2), are currently in clinical trials for COVID-19 [100]. The combination of baricitinib, a non-specific JAK inhibitor, with the anti-viral remdesivir reduced mortality and improved recovery time in hospitalized COVID-19 patients [101]. In lung cultures infected with Influenza A virus, TYK2 inhibition improved the innate immune response through IL-1B, reducing the development of secondary bacterial pneumonia [102]. Thus, TYK2 is a potential target for treatment of patients with COVID-19 or viral pneumonia to combat overactive cytokine inflammation [103].

Genetic association studies have also linked the *TYK2* gene to the risk of developing many autoimmune diseases [34]. Likewise, while *Tyk2^-/-^* mice remain viable and fertile, they are more resistant to autoimmune, allergic and inflammatory diseases [47,68,99]. A genome-wide association study (GWAS) meta-analysis, and other studies, found that the *TYK2* P1104A variant was protective against multiple autoimmune diseases, including multiple sclerosis (MS), psoriasis, inflammatory bowel disease (IBD), ankylosing spondylitis, rheumatoid arthritis (RA), systematic lupus erythematosus (SLE), and type-I diabetes (T1D), without increased susceptibility to infection (see Table 1) [31,32,33,35]. Mouse studies indicated that the *Tyk2* P1104A variant decreased response to IL-12, IL-23 and IFN-I, while in vitro mutational investigations showed that IFN-α/β, IL-6 and IL-10 signaling remained intact in *TYK2* P1104A cells [39,104]. Interestingly, a recent study describes a *TYK2* splice variant (Rs2304256) that is protective against SLE, T1D and psoriasis [34]. Rs2304256 (V362F in the FERM domain) contains exon 8, resulting in enhanced TYK2 binding to cytokine receptors and increased *TYK2* gene expression [34]. A meta-analysis of immunochip data in autoimmune diseases identified the *TYK2* SNP rs74956615 as associated with RA, T1D, and systemic sclerosis (SSc) [30]. Thus, there is a complex balance of TYK2 signaling as both a mediator of cytokine signaling important in immunity and the involvement of TYK2 in excessive inflammation and autoimmune dysfunction. In addition, TYK2 plays an important role in tumor immunosurveillance as well as inflammatory cytokine signaling in cancer development and metastases, which was recently reviewed [44].

## 4. Pro-survival Actions of TYK2 and STATs in Cancer

**Figure 3:**
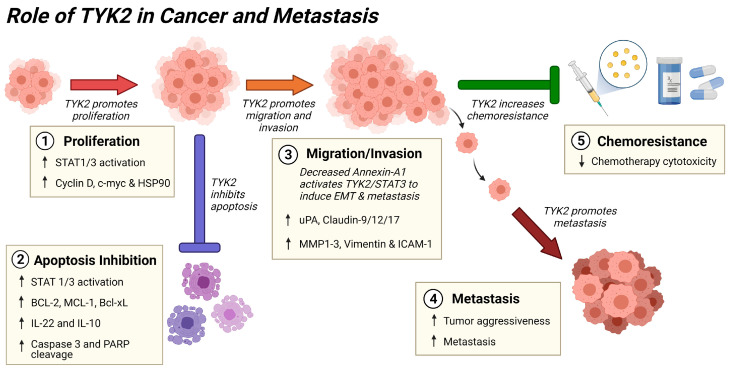
Summary of the role of TYK2 signaling in cancer progression and metastasis. TYK2 activates STATs to inhibit cancer cell apoptosis [18,20,41,106,108,109] while stimulating proliferation [20,41,106,108,113], migration, invasion [113,114,115,116,117,118], and resistance to chemotherapy [107]. These functions of TYK2 lead to increased tumor aggressiveness and metastasis [14,20,22,36,106], resulting in greater patient mortality. Arrows (↑ or ↓) indicate direction of gene expression or protein activation change. Illustration created with BioRender.com.

TYK2 has emerged as a pro-survival factor in many types of cancer through stimulation of proliferation and protection from cell death (Figure 3) [105]. In various cancers, TYK2 overexpression or GOF mutations lead to STAT1 or STAT3 activation and upregulation of anti-apoptotic proteins, including B-cell CLL/lymphoma-2 (BCL-2) and myeloid cell leukaemia-1 (MCL-1) [20,41]. Recent reports investigated the pro-survival mechanisms of TYK2 signaling using pharmacologic or genetic inhibition of TYK2 or STAT3 in various cancer cell lines and mouse tumor models [20,41,106,107]. In human ALCL cells, inhibition of TYK2 by small molecule inhibitors or genetic depletion decreased proliferation and induced apoptosis with concomitant reductions in STAT1/STAT3 activation, MCL-1, IL-22 and IL-10 [41]. Correspondingly, loss of *Tyk2* in an NPM-ALK lymphoma mouse model delayed tumor growth and prolonged overall survival [41]. Likewise, TYK2 is highly expressed in MPNST compared to lower expression in benign precursor plexiform neurofibroma tumors [20]. TYK2 deficiency reduces MPNST tumor growth through decreased proliferation and increased apoptosis mediated via lower phosphorylated STAT3 (p-STAT3) and BCL-2 with a concomitant increase in caspase 3 cleavage [20]. In esophageal cancer, *TYK2* is also overexpressed, and associated with later stages of disease and shorter patient survival [106]. The flavonoid cirsiliol blocks TYK2-STAT3 induced cell proliferation and patient-derived xenograft (PDX) esophageal cancer tumor growth, likely through decreased C-MYC, BCL-2 and MCL-1 protein levels [106]. Fibroblast growth factor-2 (FGF-2) activates TYK2 to stimulate proliferation and protect osteosarcoma cells from chemotherapeutic drugs in vitro [107]. In B-cell lymphoma cells, binding of the CD86 ligand to cytotoxic T-lymphocyte-associated antigen 4 (CTLA4) results in recruitment and phosphorylation of TYK2, which activates STAT3 to drive transcription of genes promoting tumor growth and survival [108]. Similarly, IL-10 activation of TYK2 or GOF mutations in *TYK2* promote survival of T-ALL cells through phosphorylation of STAT1, but not STAT3, and upregulation of BCL-2 [18]. In head and neck cancer cells, inhibition of STAT3 leads to cell cycle arrest and apoptosis through pro-apoptotic PARP cleavage and decreases in anti-apoptotic BCL-xL and survivin [109].

STAT transcription factors have also been widely studied for their role in carcinogenesis, as they are frequently mutated or highly activated in cancer [110]. The major oncogenic actions of TYK2 are mediated through STAT1, STAT3, and STAT5, and drugs targeting STATs are in development for cancer therapeutics [111,112]. The major cytokines with upregulated gene expression due to TYK2/STAT signaling include type I IFN, type 2 IFN, IL-15 and IL-17 [44]. Other STAT target genes are involved in cancer signaling to regulate cell proliferation (e.g., cyclin D, HSP90), apoptosis (e.g., BCL-2, MCL-1, Bcl-xL), angiogenesis (e.g., VEGF-A, FGF), metastasis (e.g., MMP1-3, Vimentin, ICAM-1) and signaling (e.g., AKT, PI3K, TNF-R2) [113].

In some normal and cancer cell types, TYK2 is involved in IFN signaling to induce apoptosis through pro-apoptotic proteins [119,120,121,122]. Type I IFN (i.e., IFN-α, IFN-β) induces apoptosis via TYK2 and STAT1/3 in normal pancreatic β-cells and hematopoietic cells [119,120,121]. In addition, the TYK2-STAT3 axis mediates β-Amyloid induction of neuronal cell death [123]. In fibrosarcoma cells, TYK2 is required for IFN-β-induced mRNA expression of tumor necrosis factor-related apoptosis inducing ligand (TRAIL), which stimulates apoptosis through death receptors [122]. Thus, the proliferative and apoptotic consequences of TYK2 actions depend on the cell type and context, including the mix of cytokines, STATs, and downstream signaling pathways.

## 5. Role of TYK2 in Metastasis

Metastasis is the primary driver of cancer mortality. Thus, it is imperative to understand the molecular mechanisms underpinning metastasis to reduce cancer deaths [124]. Epithelial-to-mesenchymal transition (EMT) is a pre-programmed process by which polarized epithelial cancer cells de-differentiate into stem cells that can migrate and invade, allowing the cells to metastasize to other locations in the body [124,125]. In a series of recent genomic, proteomic, cell-based and mouse model studies, *TYK2* has emerged as an oncogene that promotes migration, invasion and metastasis in multiple types of cancer, including stomach, MPNST, breast cancer, liver cancer, colon cancer, prostate cancer and lung cancer [14,20,22,114,115,116].

Using an RNA-seq bioinformatics approach to compare stomach adenocarcinoma and normal tissues, *TYK2* and *JAK3* mRNA expression were positively associated with tumor grade, stage, and lymph node metastasis status, and patients with high *TYK2* expression in their tumors had shorter overall survival [14]. Therefore, TYK2 and JAK3 may function as biomarkers in stomach cancer, as they are associated with tumor aggressiveness and metastasis [14]. A genomic NGS screen showed colorectal cancer metastasis to the liver contained a *TYK2* SNP (rs2304256, V362F), although the mechanism of this mutation was not investigated [22]. Likewise, a genomic NGS analysis identified activating *TYK2* mutations in MPNST [36]. In follow-up studies employing a metastatic mouse xenograft model with left ventricle tumor injection, shRNA knockdown of *Tyk2* decreased MPNST tumor burden and increased overall survival [20].

The process of invasion is critical in EMT and metastases, and is regulated in part by tight junction proteins and matrix metalloproteinases (MMP) [126]. In lung cancer, the tight junction protein claudin-12 stimulates EMT via TYK2 and STAT1 through increased cell migration and invasion [116]. Additionally, elevated tight junction proteins claudin-9 and claudin-17 stimulate migration and invasion of hepatocellular carcinoma (HCC) (liver cancer) cells through activation of the TYK2/STAT3 pathway [115,117]. Similarly, in prostate cancer cells, inhibition of *TYK2* gene expression by shRNA decreases invasion through decreased urokinase-type plasminogen activator (uPA), which is involved in the activation of MMPs that degrade extracellular matrix proteins [114,127]. Annexin A1 (AnxA1) functions as a suppressor of EMT and metastasis in breast cancer, and AnxA1 deficiency is associated with poor prognosis and metastasis [118]. Knockdown of AnxA1 induces EMT and metastasis, which is also mediated by TYK2 and STAT3 [118].

As discussed earlier, the role of TYK2 in cancer is complex, and some groups have reported an inhibitory role of TYK2 in metastases [16,42,128]. Proteomic analysis using tissue microarrays from a cohort of 70 patients with different breast pathologies revealed that TYK2 was down-regulated during metastasis to the regional lymph node [16]. In agreement with this, 4T1 breast cancer cells with TYK2 deficiency demonstrate enhanced tumor growth and metastasis [42]. Higher levels of TYK2 were also associated with positive prognosis and immune infiltrate in lung adenocarcinoma [128]. Taken together, all these data suggest a cell type contextual role of TYK2 in metastasis.

## 6. Pharmacologic Inhibition of TYK2 for Treatment of Cancer and Metastasis

Selective inhibitors of JAKs (JAK1-3/TYK2) were originally developed as therapeutics for autoimmune diseases (e.g., rheumatoid arthritis, ankylysing spondylitis and ulcerative colitis), as well as organ transplantations, due to their ability to suppress the immune response (Table 2) [129,130]. In 2011, the pan-JAK inhibitor tofacitinib was the first JAK inhibitor to be FDA-approved to treat rheumatoid arthritis [131,132]. Tofacitinib targets JAK3 and JAK1, with some effect on JAK2 and a small component on TYK2 [129]. Second generation JAKinibs, including baricitinib and upadacitinib, were developed to be more selective and reduce significant side effects [129]. The use of JAK inhibitors (JAKinibs) was expanded to cancer with the advent of ruxolitinib, a selective JAK1/JAK2 inhibitor approved to treat the hematological cancers myelofibrosis and polycythemia vera [133]. However, the class of JAK inhibitors, including tofacitinib, baricitinib, and upadacitinib, have recently undergone further regulatory scrutiny for serious adverse events, with restrictions now placed on indications and dosage [130].

In an effort to lessen side effects while improving efficacy, TYK2 inhibitors (TYKinibs) have become a recent focus in drug discovery [147,148,149]. Progress in the creation of specific inhibitors against the different members of the JAK family has been hindered by high protein homology within the catalytic JH1 domain [150]. The kinase JH1 domain is the target of most JAKinibs, including the first generation selective TYK2 inhibitors, some of which show promise as cancer therapeutics [151,152]. A second-generation TYK2 inhibitor, deucravacitinib (BMS-986165), has recently completed Phase III clinical trials for plaque psoriasis through blockade of the IL-12, IL-23 and type I IFN pathways [150,153]. Unlike previous JAKinibs, the highly selective deucravacitinib is directed against the regulatory JH2 pseudokinase domain of TYK2, and does not inhibit JAK1-3 at clinical therapeutic doses. Thus, deucravacitinib and other second generation TYKinibs in development are reported to have better safety profiles than older JAKinibs [141]. Preclinical studies in cell lines and mice have expanded the use of TYK2 inhibitors to cancer, with the TYKinibs NDI-031301, SAR-20347 and SAR-20351 inducing regression of T-ALL and solid cancer tumors [154,155]. These first and second-generation TYK2 inhibitors present an exciting opportunity for targeted cancer therapy.

However, as is the case for many targeted therapies, treatment failure can occur in the setting of intrinsic resistance to JAK or TYK2 inhibitory drugs, or development of resistance to these drugs over time [156,157]. For example, approximately half of myeloproliferative neoplasm (MPN) patients become resistant to long-term ruxolitinib treatment after two to three years, resulting in poor outcomes [158,159,160]. In vitro studies attribute ruxolitinib resistance in hematological cancers to either sustained JAK/STAT activation due to heterodimerization and transactivation of various JAK family members, to secondary mutations acquired in the *JAK2* kinase domain that reduce drug binding, or to activation of the RAS, ERK and Akt pathways [161,162,163,164]. Many MPN patients also harbor mutations in genes involved in epigenetic regulation, in addition to a mutated *JAK2 V617F* [156]. Similarly, despite an initial therapeutic response, ALL cells expressing the *MYB-TYK2* fusion protein eventually become insensitive to the JAK/TYK inhibitor cerdulatinib after prolonged incubation due to a mutation in the *TYK2* kinase domain and overactive JAK/STAT [157].

Development of combination therapies is one approach to combat resistance. Recent studies have evaluated several combination therapies in attempts to improve overall efficacy or overcome resistance to TYKinibs/JAKinibs or other cancer drugs [156,157,164]. In ALL *MYB-TYK2* cells with acquired cerdulatinib resistance, histone deacetylase inhibitor (HDACi) therapy proved efficacious in blocking proliferation [157]. Likewise, heat shock protein 90 (HSP90) inhibitors combined with JAK inhibitors (e.g., ruxolitinib, TG101209) overcome JAKinib resistance in leukemia and MPN cells [165,166]. Indeed, response to combination therapy consisting of ruxolitinib with the HSP90 inhibitor, HDACi, and/or chemotherapy was superior to monotherapy in both preclinical murine studies, as well as in early studies in patients with leukemia [156]. Ruxolitinib resistance can also be bypassed with subsequent treatment with another JAK2 inhibitor, including fedratinib, pacritinib and momelotinib [167]. In addition, TYK2 and JAK inhibitors are starting to be evaluated in combination with chemotherapy drugs or other pathway inhibitors. For example, the selective TYK2 inhibitor SAR-20351 acts synergistically in combination with the chemotherapeutic 5-fluorouracil (5-FU) or anti-CTLA-4 therapy in colon cancer mouse models, or with everolimus, an mTOR kinase inhibitor, in a renal cell cancer mouse model [155]. Similarly, while colorectal cancer cells can evade inhibition of JAK1/2 through aberrant activation of the RAS-MEK-ERK pathway, combination therapy with the JAK1/2 inhibitor momelotinib sensitizes these cells to trametinib, a MEK inhibitor [168]. 

## 7. Conclusions and Perspectives

Personalized, or precision, medicine has increasingly become a valuable tool in clinical diagnostics to evaluate potential therapeutic options for individual cancer patients [169]. Based on genetic mutations, gene expression or protein level changes in patient tumors for known oncogenes or tumor suppressors, personalized medicine can aid in prognostication, as well as predict responses to anti-cancer agents [170]. Given the identification of TYK2 as a potential biomarker for numerous types of cancer, evaluation of patient tumors for *TYK2* mutations or expression levels may help determine patient prognosis. In addition, with the development of multiple pharmacologic inhibitors of TYK2 and JAKs, those tumors that are TYK2-positive may be viable candidates for TYKinib/JAKinib targeted therapies. Future pre-clinical studies should be geared at evaluating therapeutic interventions with TYK2 inhibitors as single agents, or in combination therapy to pave the way for biomarker-driven clinical trials.

## Figures and Tables

**Table 1:** Genomic, transcriptomic and proteomic studies identify TYK2 mutations and expression in cancer.

Study Type	Type of Screen for Discovery	SNP, Variant, Fusion Protein	Disease	Tyk2 Activity	Effect on Disease	Ref.
*Proteomic*	DAMA staining screen	nd	Breast Cancer	High TYK2 levels	nd	[10]
	Mass Spectrometry	nd	Squamous Cervical Cancer	High TYK2 levels	nd	[11]
	Phospho-tyrosine Mass Spectrometry	nd	Colorectal Cancer	High p-TYK2 levels with HGF stimulation	↑ Proliferation	[12]
	Mass Spectrometry	Splice variant (E971fsX67)	Brain Hematopoietic cancer	Inactivating TYK2 mutations	↑ Disease risk	[15]
	Mass Spectrometry	nd	Breast cancer metastasis to regional lymph nodes	Low TYK2 levels	nd	[16]
	Phospho-tyrosine Mass Spectrometry	nd	Metastatic Prostate Cancer	High p-TYK2 levels	nd	[13]
*Transcriptomic*	RNA-Seq	nd	Stomach Adenocarcinoma	High TYK2 levels	Prognostic Biomarker	[14]
	RNAseq Screen	NFκB2-TYK2 PABPC4-TYK2 fusion proteins	ACLC	Constitutively Active TYK2	↑ Proliferation ↑ Disease risk	[17]
	RNAi screen	Point Mutation in FERM, JH2, Kinase Domain	T-ALL	GOF mutations	↑ Proliferation ↓ Apoptosis ↑ Disease risk	[18]
	Whole Transcriptome Sequencing	NPMI-TYK2 fusion protein	T-cell lymphoma	Constitutively active TYK2	↑ Disease risk	[19]
*Genomic*	NGS	rs34536443 (P1104A)	MPNST sarcomas	High TYK2 levels	↑ Proliferation ↓ Apoptosis ↑ Disease risk	[20]
	NGS	TYK2 mutation	Sarcomas	nd	↑ Disease risk	[21]
	NGS	rs2304256 (V362F)	Colorectal cancer metastasis	nd	↑ Disease risk	[22]
	Whole Exome Sequencing	G761V P760L (in JH2 domain)	Pediatric ALL	Constitutively active TYK2	↑ Disease risk	[23,24]
	NGS	Multiple kinase activating mutations MYB-TYK2	Ph-like ALL	High TYK2 activity	↑ Disease risk	[25,26]
	High Throughput Sequencing	rs2304255 (G363S)	AML	nd	nd	[27]
	Amplicon-based NGS	TYK2 variants with lower TYK2 gene expression	B-ALL	Catalytic LOF	↑ Disease risk	[28]
*Genomic*	GWAS	rs74956615	COVID-19	High TYK2 levels	↑ Severe disease risk	[29]
	Immunochip meta-analysis	rs74956615	RA, T1D, SSc	nd	↑ Disease risk	[30]
	GWAS, Immunochip	rs34536443 (P1104A)	MS, IBD, AS, psoriasis	nd	Protective	[31,32,33]
	Immunochip	rs9797854 rs12720356 (I684S)	MS, IBD, AS, psoriasis	nd	Variable depending on disease	[31,32,33]
	eQTL analysis	rs2304256 (V362F) rs12720270	SLE, T1D, psoriasis	Increased TYK2 binding due to exon 8	Protective	[34]
	Immunochip & Exomechip genotyping, Exon Sequencing	rs34536443 (P1104A) rs35018800 (A928V) rs12720356 (I684S)	RA, SLE, IBD		Protective	[35]

Arrows (↑ or ↓) indicate increase or decrease, respectively, in proliferation, apoptosis or disease risk. Ref. = References, DAMA = dissociable antibody microarray staining screen, nd = not determined, p-TYK2 = phosphorylated TYK2, HGF = hepatocyte growth factor, RNA-Seq = RNA-Sequencing, NGS = next generation sequencing, GOF = gain of function, LOF = Loss of function, ALL = Acute lymphoblastic leukemia, T-ALL = T-cell ALL, AML = Acute myeloid leukemia, ACLC = Anaplastic large cell lymphoma, Ph-like ALL = Philadelphia chromosome-like ALL, RNAi = RNA interference, NPMI = nucleophosmin, NFKB2 = nuclear factor of kappa light polypeptide gene enhancer in B cells 2, PABPC4 = poly(A) binding protein cytoplasmatic 4, MYB-TYK2 = v-myb avian myeloblastosis viral oncogene homolog, GWAS = genome-wide association study, MS = multiple sclerosis (MS), IBD = inflammatory bowel disease, AS = ankylosing spondylitis, RA = rheumatoid arthritis, SLE = systematic lupus erythematosus, T1D = Type-I diabetes, SSc = systemic sclerosis, eQTL = expression quantitative trait locus.

**Table 2:** Pharmacologic Inhibitors of JAKs/TYK2 (FDA-approved/In Clinical Trials).

Drug Name (Generic/Commercial)	Targeted JAKs/TYK2	Targeted Domains	FDA-Approval/Disease	References
Ruxolitinib (Jakafi)	**JAK1/2** *TYK2* * JAK3*	JH1	GVHD, Myelofibrosis, Polycythemia COVID-19 (Phase 3)	[133,134,135,136,137]
Baricitinib (Olumiant)	**JAK1/2**	JH1	Approved for Moderate/Severe RA COVID-19 (Emergency Use with Remdesvir)	[129,138]
Tofacitinib (Xeljanz)	**JAK1/3** *JAK2* * TYK2*	JH1	Approved for RA, Psoriatic Arthritis, Moderate/Severe Ulcerative Colitis, Polyarticular Course Juvenile Idiopathic Arthritis	[129]
Upadacintinib (Rinvoq)	**JAK 1/2** *JAK3* * TYK2*	JH1	Approved for Moderate/Severe RA	[129,139]
Fedratinib (Inrebic)	**JAK 2** *JAK 1/3*	Substrate binding site	Approved for Myelofibrosis	[140]
Deucravacitinib(BMS-986165)	**TYK2**	JH2	Plaque Psoriasis (Phase 3)	[141]
Momelotinib	**JAK 1/2**	ATP-Competitive inhibitor	Myelofibrosis (Phase 3, Fast-track designation)	[142]
Filgotinib (Jyseleca)	**JAK 1**	JH1	RA, Ulcerative Colitis (Phase 3)	[139]
Cerdulatinib	**JAK1** **JAK2** **JAK3** **TYK2**	SYK/JAK Kinase Inhibitor	PTCL (Phase 2B, Orphan drug designation)	[143,144]
Tyk2-IN-8, compound 10 (PF-06826647)	**JAK 1/2** **TYK2**	JH1	Moderate/Severe Psoriasis (Phase 2)	[145]
AZD1480	**JAK2** *JAK3* * TYK2* * JAK1*	ATP-Competitive inhibitor	Solid Tumors (Phase 1)	[146]

Primary JAK/TYK2 targeted proteins in bold and targets with less affinity in italics. Clinical trial phase indicated in parentheses. GVHD = graft-versus-host disease, RA = rheumatoid arthritis, PTCL = peripheral T-cell lymphoma.
